# Cross-sectional and longitudinal evaluation of heart-to-brachium pulse wave velocity for cardiovascular disease risk

**DOI:** 10.1038/s41440-024-01805-5

**Published:** 2024-08-01

**Authors:** Jun Sugawara, Hirofumi Tanaka, Akira Yamashina, Hirofumi Tomiyama

**Affiliations:** 1 Human Informatics and Interaction Research Institute, National Institute of Advanced Industrial Science and Technology, Tsukuba, Japan; 2 Department of Kinesiology and Health Education, The University of Texas at Austin, Austin, TX USA; 3 Department of Nursing, Kiryu University, Midori, Japan; 4 Department of Cardiology, Tokyo Medical University, Shinjuku City, Japan

**Keywords:** Arterial stiffness, Framingham risk score, A receiver operating characteristic curve

## Abstract

Heart-brachium pulse wave velocity (hbPWV) is a promising measure of arterial stiffness including the proximal aorta. To characterize age-associated changes and the clinical utilities of hbPWV, we evaluated the impacts of age and cardiovascular disease (CVD) risks on hbPWV cross-sectionally (*N* = 7868) and longitudinally (*N* = 3710, followed by 9.1 ± 2.0 years). hbPWV were obtained using two validated equations for arterial path length (with and without considering age-related aortic elongations). Brachial-ankle pulse wave velocity (baPWV) was used as a comparative measure. Repeated-measures correlation (rmcorr) and regression analyses were used to characterize associations of PWVs with age and Framingham’s general CVD risk score (FRS). In the cross-sectional study, hbPWVs derived by both equations showed stronger correlation with age (r = 0.746 ~ 0.796) and FRS (r = 0.714–0.749) than baPWV (r = 0.554 and r = 0.643). Furthermore, hbPWVs correlated with FRS even after controlling for age (r = 0.260 ~ 0.269, P < 0.0001). In the longitudinal study, hbPWVs demonstrated significantly higher rmcorr coefficient with age than baPWV (r_rm_=0.439–0.511 vs. 0.307, *P* < 0.0001). Across the adult lifespan, age-related increases in hbPWVs were almost consistent, starting from young adults, while baPWV displayed accelerated increases with age. A receiver operating characteristic curve analysis indicated that hbPWVs depicted more robust ability to stratify general CVD risk compared with baPWV (AUC = 0.896–0.913 vs. 0.833, *P* < 0.0001). The results of the follow-up study were consistent with the findings of the cross-sectional investigation. Our findings suggest that hbPWV undergoes a linear augmentation with age, commencing from an early adult life stage onward, rendering it a potential marker for discerning CVD risk.

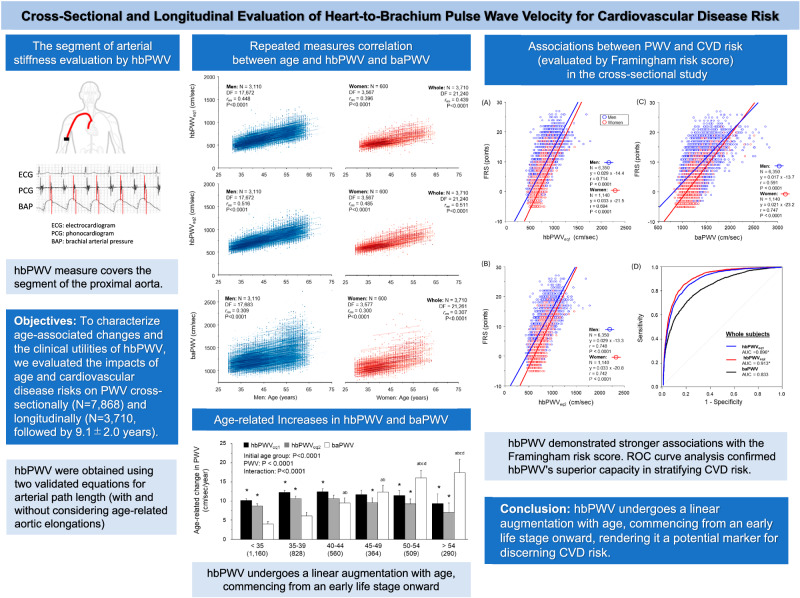

## Introduction

The arterial system has a pivotal role called the “Windkessel” function. The arterial wall expands and recoils repeatedly with cardiac ejection from the left ventricle (LV), which minimizes energy wastage and maintains extra component of hydraulic load as low as possible [1–3]. Because of the prominent elastic and viscous properties and the anatomical location directly connected to the LV, the proximal aorta is considered the most important segment for the arterial buffering function. The stiffening of the arterial wall with advancing age is particularly pronounced in the proximal aorta [4–6]. It contributes importantly to elevated risks of cardiovascular diseases (CVD), cardiovascular events, and all-cause mortalities [7–10]. Despite recognizing that the assessment of proximal aortic stiffness would be valuable, few methodologies (e.g., magnetic resonance imaging [MRI]) are targeted for evaluations in the proximal aorta [5, 11].

Pulse wave velocity has become the reference standard modality for assessing arterial stiffness due to a compelling association between elevated carotid-femoral pulse wave velocity (cfPWV) and risks for the development of CVD [12–15]. Additionally, brachial-ankle pulse wave velocity (baPWV) is also associated with an increased risk of total cardiovascular events and all-cause mortality [13, 15, 16] and has garnered increasing acceptance [17, 18]. These measures of arterial stiffness, baPWV and cfPWV, are significantly associated with each other as they reflect overlapping regional arterial properties and functions [19–21]. However, neither PWV measure covers the segment of the proximal aorta. In this context, heart-to-brachium pulse wave velocity (hbPWV) is a promising method [6, 22]. Pulse transit time can be evaluated easily by simultaneous recordings of the heart sound or ECG and brachial arterial pulse waves recorded with the high-fidelity sensor embedded in the blood pressure cuff. As an initial step to evaluate this technique, we measured the arterial path length from the heart and the brachium using MRI and constructed formulas for estimating arterial path length [22]. A subsequent cross-sectional study employing the arterial path length estimation showed that hbPWV displayed one of the largest age-related increases compared with other measures of arterial stiffness, including cfPWV and baPWV [6]. These results were confirmed by a 10-year follow-up investigation [6]. However, due to the small sample size and the lack of clinical evidence, a larger-scale study focusing on clinical evidence is needed for hbPWV to gain more acceptance.

In the present study, we utilized both cross-sectional and longitudinal study designs to characterize age-related changes in hbPWV in a large cohort of middle-aged Japanese workers [23, 24]. For comparison purposes, baPWV was also assessed since this measure of arterial stiffness has been widely studied and implemented, particularly in Japanese populations. Additionally, clinical utilities of hbPWV were evaluated against general CVD risks evaluated with Framingham’s risk factors [25].

Point of view

- Clinical relevance
  hbPWV undergoes a linear augmentation with age, commencing from an early adult life stage onward, rendering it a potential marker for discerning CVD risk.
- Future direction
  The sensitivity and specificity of predicting future CVD events using hbPWV necessitate validation through prospective studies.
- Consideration for the Asian population
  As many Asian countries are experiencing rapid growth of the aging population, arterial stiffness, an indicator of biological aging, can have substantial public health implications for Asian populations. hbPWV may be a promising measure of arterial stiffness that may improve the early detection capabilities of CVD risk.

## Methods

### Design and participants

The present study was initiated on the employees working at the headquarters of a single large Japanese construction company in 2000 when the Occupational Health and Safety Law in Japan mandated annual health checkups for large companies. All company employees had to undergo annual health checkups. Participants underwent a physical examination, phlebotomy, anthropometry, four-extremities blood pressure (BP) and arterial stiffness (via PWV) measurements. Individuals with risk factors for CVD were advised to visit the health care center within the company. They received advice for therapeutic lifestyle modifications by health professionals following the Japanese guidelines [26–29]. Individuals requiring medications had appropriate drugs prescribed at the health care center or other clinics. The record of hbPWV measurement was not available till 2003 because of technical reasons. The available data from 2003 to 2013 were analyzed for the present study. Inclusion criteria were <75 years of age, ankle-brachial index ≥0.9, and no history of CVD. Verbal informed consent was obtained from the study participants before participating in this study. The study was conducted with the approval of the Ethics Guidelines Committee of Tokyo Medical University (No. SH3450). Some data from this study have been published elsewhere to examine the longitudinal association of arterial stiffness (baPWV) with cardiovascular risk status [23, 24].

### Risk factors for cardiovascular disease

All the blood samples were obtained in the morning after overnight fasting. Plasma glucose concentration and serum concentrations of triglyceride, total cholesterol, and HDL-cholesterol were measured with standard enzymatic methods (Falco Biosystems Company, Ltd., Tokyo, Japan) (20). Diabetes was defined as ≥126 mg/dL of fasting glucose and/or use of insulin or oral hypoglycemic medications. Antihypertensive medication use was based on self-report. Cigarette smoking status was ascertained by self-report. Framingham’s general CVD risk score (FRS) was evaluated by sex-specific multivariable risk functions (“general CVD” algorithms) incorporated age, total and high-density lipoprotein cholesterol, systolic blood pressure, pharmacological treatment for hypertension, smoking, and diabetes status [25].

### Cardiovascular measurements

Heart rate, BP, and PWV were measured noninvasively with the automated cardiovascular screening device (Form/ABI, Colin Company, Ltd., Komaki, Japan) equipped with an electrocardiogram, phonocardiogram, and four-extremity BP cuffs involving air-plethysmograph [20, 21, 23] after at least 5 min in the supine position in an air-conditioned room (maintained at 24°C) allocated exclusively for this study. Electrocardiographic electrodes were placed on both wrists, and a microphone for the phonocardiogram was placed on the left chest. Bilateral brachial and posterior-tibial arterial pressure waveforms were recorded for 10 s by occlusion cuffs connected to air-plethysmographic sensors wrapped on both arms and ankles. PWV is calculated from the arterial path length divided by the pulse transit time.

Pulse transit times were determined from the time delays from the proximal (i.e., the right brachial artery) to the distal (i.e., both posterior-tibial arteries) “foot” waveforms (Tba) for baPWV and from the second heart sound to the dicrotic notch on the arterial waveforms at the right brachium (Thb) for hbPWV [6, 22]. hbPWV was measured only on the right arm due to the default settings of the automated cardiovascular screening device. Arterial path lengths for baPWV (Lba) and hbPWV were calculated from the following equations [20, 22]:

$${{{{\rm{Lba}}}}}({{{{\rm{cm}}}}})=0.59\times {{{{\rm{height}}}}}\left({{{{\rm{cm}}}}}\right)+14.40$$

$${{{{{\rm{Lhb}}}}}}_{{{{{\rm{eq}}}}}1}({{{{\rm{c}}}}}{{{{\rm{m}}}}})= 3.79\times {{{{\rm{sex}}}}}({{{{\rm{male}}}}}=1{{{{\rm{;female}}}}}=0)\\ + \, 0.09\times {{{{\rm{height}}}}}\left({{{{\rm{cm}}}}}\right)+31.65$$

$${{{{{\rm{Lhb}}}}}}_{{{{{\rm{eq}}}}}2}({{{{\rm{c}}}}}{{{{\rm{m}}}}})= \, 	 1.65\times {{{{\rm{sex}}}}}({{{{\rm{male}}}}}=1,{{{{\rm{female}}}}}=0)\\ 	 +0.14\times {{{{\rm{age}}}}}({{{{\rm{year}}}}})+0.25\times {{{{\rm{height}}}}}({{{{\rm{cm}}}}})+1.48$$

Lhb_eq2_ incorporates the age-related elongation of the proximal aorta (e.g., the ascending aorta and aortic arch) [30–32]. Consequently, baPWV and two hbPWVs were calculated as Lba/Tba and Lhb_eq_/Thb (using both equations), respectively. The higher value of either the right or left sides was used for representative baPWV to avoid underestimating CVD risk. Ankle-brachial index (ABI=ankle SBP/brachial SBP) was evaluated for screening for possible peripheral artery disease [33]. The left ventricular ejection time (ET, the interval from the onset of upstroke to the dicrotic notch on the brachial pressure waveform) and the pre-ejection period (PEP, calculated by subtracting the ET from the time between the Q wave of ECG to the second heart sound) were measured. The ratio of PEP to ET was calculated as an index of the left ventricular systolic function [34].

Before this study, we confirmed the day-to-day reproducibility of Thb (coefficient of variation = 4.1%) to be equivalent to those of heart rate, blood pressure, and baPWV (3.2, 4.7, and 3.4%, respectively).

### Statistical analyses

Univariate and partial correlation analyses were used to determine the relationship between PWV and variables related to CVD risk. A stepwise regression analysis was used to determine independent correlates of interest variables. In the cross-sectional study, only data from the first visit were used for correlation analysis to avoid double counting the same participants. In the longitudinal study, data of participants who attended ≥4 examinations for ≥5 years were analyzed. Regarding the age-related changes in PWV, we employed the repeated measures correlation (rmcorr), a statistical technique for determining the common within-individual association for paired measures assessed on more occasions for multiple individuals [35]. This method was chosen because conventional correlation analysis, when applied to non-independent observations such as repeated-measures aggregated data, can produce biased results due to the violation of independence and/or differing patterns between subjects versus within subjects [35]. The age-related changes in PWV were characterized by linear regression analyses and the repeated-measures correlation (rmcorr) using the R package [35]. Two correlation coefficients were compared by Fisher’s z-transformation. The slope of the regression line between age and PWV was compared among groups categorized by the initial age of participation (< 35, 35–39, 40–44, 45–49, 50–54, and ≥55 years) as well as PWVs by the repeated-measures ANOVA. Sub-analysis to determine the influence of sex on age-related changes in PWVs was also performed by repeated measures ANOVA (PWVs × the initial age of participation × sex). Data obtained at the first (oldest) and final (latest) examinations were selected for determining associations between PWVs and FRS in the longitudinal study. A receiver operating characteristic (ROC) curve analysis was performed to confirm whether hbPWV stratifies a more than moderate (>10% of 10-year risk) general CVD risk evaluated by “general CVD algorithms” [25] in the cross-sectional and longitudinal study designs. Cutoff points in the ROC curve were defined by the Youden index. The diagnostic accuracy of PWVs was evaluated by comparing AUC via DeLong’s test [36]. All the analyses were conducted with RStudio (2023.09.1 Build 494, Posit Software, PBC) and Statistica (ver. 13.5.0.17, TIBCO Software Inc.). The *P* values < 0.05 were considered to denote statistical significance.

## Results

A flowchart of the selection process for the participants is depicted in Supplementary Fig. 1. A total of 8,035 employees took annual health checkups at least once throughout the observation period. One participant aged ≥75 years, 123 participants with CVD history, 42 who had ABI < 0.9, and 1 with CVD history and ABI < 0.9 at the initial examination were excluded. Thus, a total of 7868 participants, including untreated patients with hypertension (*N* = 1199) and diabetes mellitus (*N* = 208), were analyzed for the cross-sectional analysis (Table 1). All subjects could be recorded right brachial arterial pressure waveforms, indicating no stenosis at the right arm. No patient with aortic disease was included (determined by the self-report).

**Table 1:** Subjects’ characteristics in the cross-sectional and longitudinal studies

	Cross-sectional study	Longitudinal study	
Variables		Initial examination	Final examination
N, men/women	6683 / 1185	3110/600	3110/600
Age, years	42.3 ± 10.3	40.5 ± 8.6	48.5 ± 8.6*
Height, cm	169.2 ± 7.5	169.5 ± 7.4	169.1 ± 7.5
Weight, kg	67.7 ± 12.0	67.8 ± 11.9	68.4 ± 12.1
BMI, kg/m^2^	23.6 ± 3.3	23.5 ± 3.3	23.8 ± 3.3
Total cholesterol, mg/dl	200.7 ± 34.7	199.8 ± 34.8	212.1 ± 33.8
LDL cholesterol, mg/dl	122.2 ± 31.4	116.8 ± 31.9	122.3 ± 31.1
HDL cholesterol, mg/dl	59.3 ± 14.8	59.3 ± 14.7	66.2 ± 16.9
Triglyceride, mg/dl	121.5 ± 101.2	118.2 ± 95.2	118.6 ± 89.6
Blood glucose, mg/dl	91.8 ± 16.9	90.8±12.9	90.3 ± 14.2
HbA1c, %	5.1 ± 0.6	5.0 ± 0.5	5.6 ± 0.7
FRS, points	7.4 ± 6.5	6.5 ± 5.9	9.4 ± 5.8
Heart rate, beat/min	66 ± 10	66 ± 10	65 ± 10
PEP, msec	97 ± 12	96 ± 12	101 ± 12
ET, msec	286 ± 21	286 ± 20	288 ± 21
PEP/ET, ratio	0.34 ± 0.05	0.34 ± 0.05	0.35 ± 0.05
Systolic BP, mmHg	127 ± 17	126 ± 15	123 ± 15
Diastolic BP, mmHg	77 ± 12	76 ± 11	77 ± 11
baPWV, cm/sec	1308 ± 223	1269 ± 181	1343 ± 222
hbPWV, cm/sec	740 ± 156	715 ± 134	806 ± 145
BP treatment, n (%)	949 (12.1)	154 (4.1)	519 (14.0)
DM treatment, n (%)	271 (3.4)	37 (1.0)	141 (3.8)
HL treatment, n (%)	451 (5.7)	49 (1.3)	236 (6.4)
Smoking habit, n (%)	3,894 (49.5)	1806 (48.7)	1806 (48.7)
Observation period, years	4.5 ± 4.0	9.1 ± 2.0	
Numbers of participation, n	4.1 ± 2.9	6.7 ± 1.7	

Data are mean and SD

*BMI* body mass index, *LDL* low-density lipoprotein, *HDL* high-density lipoprotein, *HbA1C* hemoglobin A1C, *FRS* Framingham risk score, *PEP* pre-ejection period, *ET* left ventricular ejection time, *BP* blood pressure, *baPWV* brachial-ankle pulse wave velocity, *hbPWV* heart-brachium pulse wave velocity, *DM* diabetes mellitus, *HL* hyperlipidemia

Among them, 3,710 participants (46.2%) met the inclusion criteria for the longitudinal analysis (attended ≥4 examinations for ≥5 years). Three hundred seventy-eight participants did not have FRS data due primarily to no phlebotomy. The average ages of the participants at the initial (oldest) and final (latest) tests were 40.5 ± 8.6 and 48.5 ± 8.6 years. The average duration and number of health checkups were 9.1 ± 2.0 years and 6.7 ± 1.7 times, respectively. Untreated patients with hypertension (*N* = 506 and 357) and diabetes mellitus (*N* = 72 and 107) were included in the initial and final experiments. All subjects could be recorded right brachial arterial pressure waveforms, indicating no stenosis at the right arm. No patient with aortic disease was included.

### Aging-related increases in PWV

**Fig. 1:**
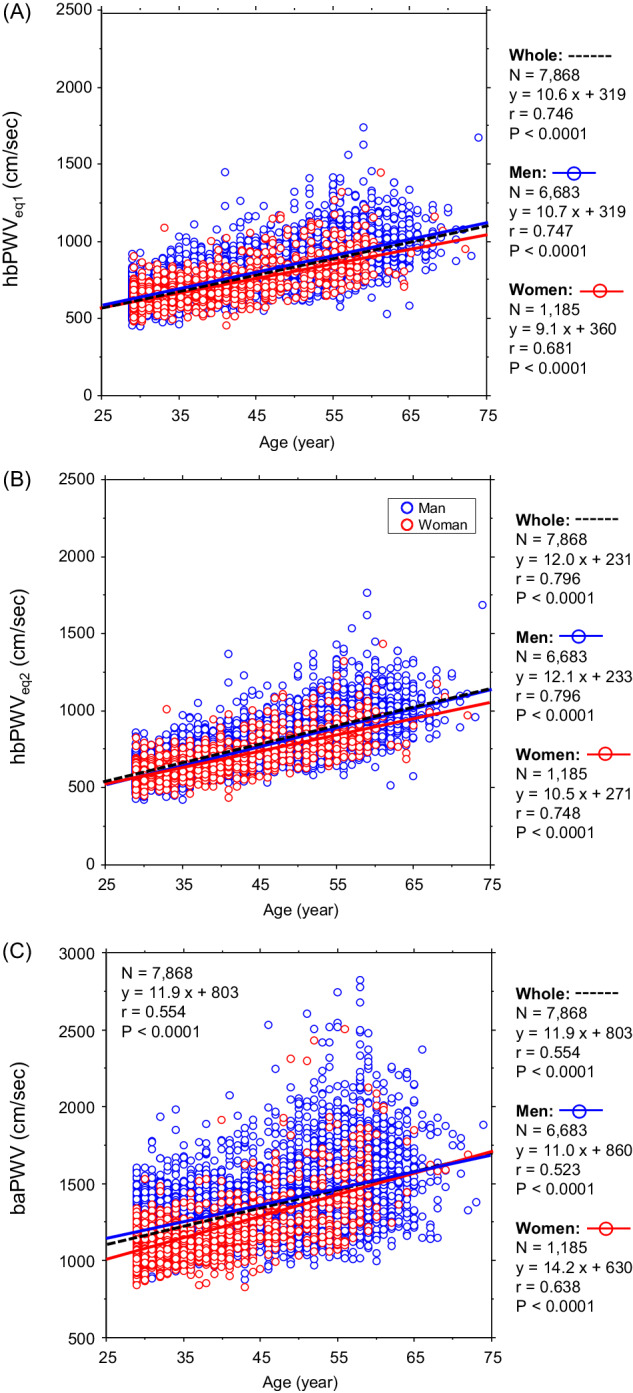
Simple correlation analyses between age and pulse wave velocity (PWV) indices in the cross-sectional study (Panel **A**: hbPWVe_q1_; Panel **B**: hbPWV_eq2_; Panel **C**: baPWV)

In the cross-sectional observation, hbPWV_eq1_ showed strong linear associations with age in men (r = 0.747, *P* < 0.0001), women (r = 0.681, *P* < 0.0001), and whole participants (r = 0.746, *P* < 0.0001) (Fig. 1A). hbPWV_eq2_ also showed strong linear associations with age in men (r = 0.796, *P* < 0.0001), women (r = 0.748, *P* < 0.0001), and whole participants (r = 0.796, *P* < 0.0001) (Fig. 1B). These correlation coefficients were significantly larger than those of baPWV (men: r = 0.523, *P* < 0.0001; women: r = 0.638, *P* < 0.0001; whole participants: r = 0.554, *P* < 0.0001) (Fig. 1C). After adjusting for the use of drugs, all correlations remained significant (via partial correlation analysis) in hbPWV_eq1_ (men: ρ = 0.747, women: ρ = 0.641, whole participants: ρ = 0.720, *P* < 0.0001 for all), hbPWV_eq2_ (men: ρ = 0.796, women: ρ = 0.716, whole participants: ρ = 0.776, *P* < 0.0001 for all), and baPWV (men: ρ = 0.475, *P* < 0.0001; women: ρ = 0.583, *P* < 0.0001; whole participants: ρ = 0.503, *P* < 0.0001).

Contributions to the age-related change in PWVs were determined between the arterial path length and pulse transit time using a stepwise regression analysis. In the cross-sectional study, T_hb_ and T_ba_ were the primary independent correlates of hbPWV_eq1_ (β = –0.92), hbPWV_eq2_ (β = –0.95), and baPWV (β = –0.95), explaining 84%, 89% and 89% of their total variance. Likewise, in the longitudinal study, changes in T_hb_ and T_ba_ were major independent correlates of changes in hbPWV_eq1_ (β = –0.93), hbPWV_eq2_ (β = –0.91), and baPWV (β = –0.94), explaining 87%, 83%, and 89% of their total variance.

**Fig. 2:**
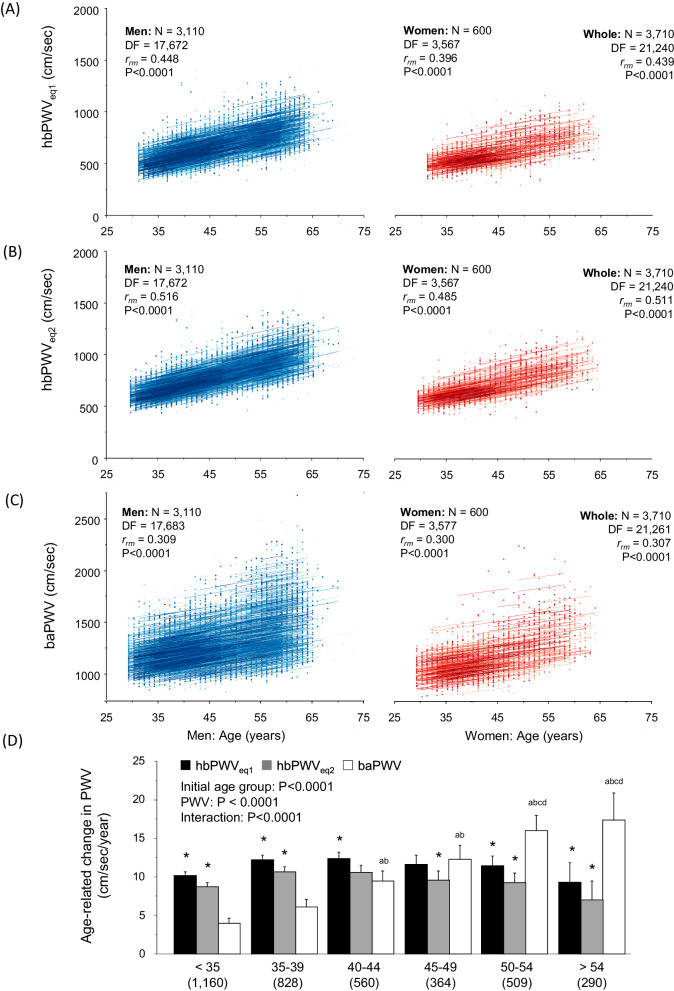
Repeated measures correlation plots between age and pulse wave velocity (PWV) indices (Panel **A**: hbPWVe_q1_; Panel **B**: hbPWV_eq2_; Panel **C**: baPWV). Lines represent intra-individual linear regression lines for each participant. DF, degrees of freedom; r_rm_, the coefficient of repeated measures correlation. Panel (**D**) presents the comparison of the age-related regression slopes of PWVs. (a) *P* < 0.0001 vs. <35 years of the initial age group. (b) *P* < 0.0001 vs. 35-39 years of the initial age group. (c) *P* < 0.0001 vs. 40–44 years of the initial age group. (d) *P* < 0.0001 vs. 45-49 years of the initial age group. *P < 0.0001 vs. baPWV. Error bars indicate 95% confidential interval

In the longitudinal observation, hbPWV_eq1_ and hbPWV_eq2_ showed significant rmcorr coefficients with age in men (r_rm_ = 0.448 and 0.516, *P* < 0.0001 for both), women (r_rm_ = 0.396 and 0.485, *P* < 0.0001 for both), and whole participants (r_rm_ = 0.439 and 0.511, *P* < 0.0001 for both) (Fig. 2A, B). These r_rm_ were significantly higher than those of baPWV (men: r_rm_ = 0.309; women: r_rm_ = 0.300; whole participants: r_rm_ = 0.307, *P* < 0.0001 for all) (Fig. 2C).

Figure 2D depicts the slopes of the regression line between age and PWVs among groups categorized by the initial age of participation. Across the age span, age-related increases in hbPWV_eq1_ and hbPWV_eq2_ were consistent, starting from young adults, while baPWV displayed accelerated increases with age. Sub-analysis to determine the influence of sex on age-related changes in PWVs showed no significant sex differences on the interaction between PWV and the initial age group (*P* = 0.316).

### Relation to CVD risk

**Fig. 3:**
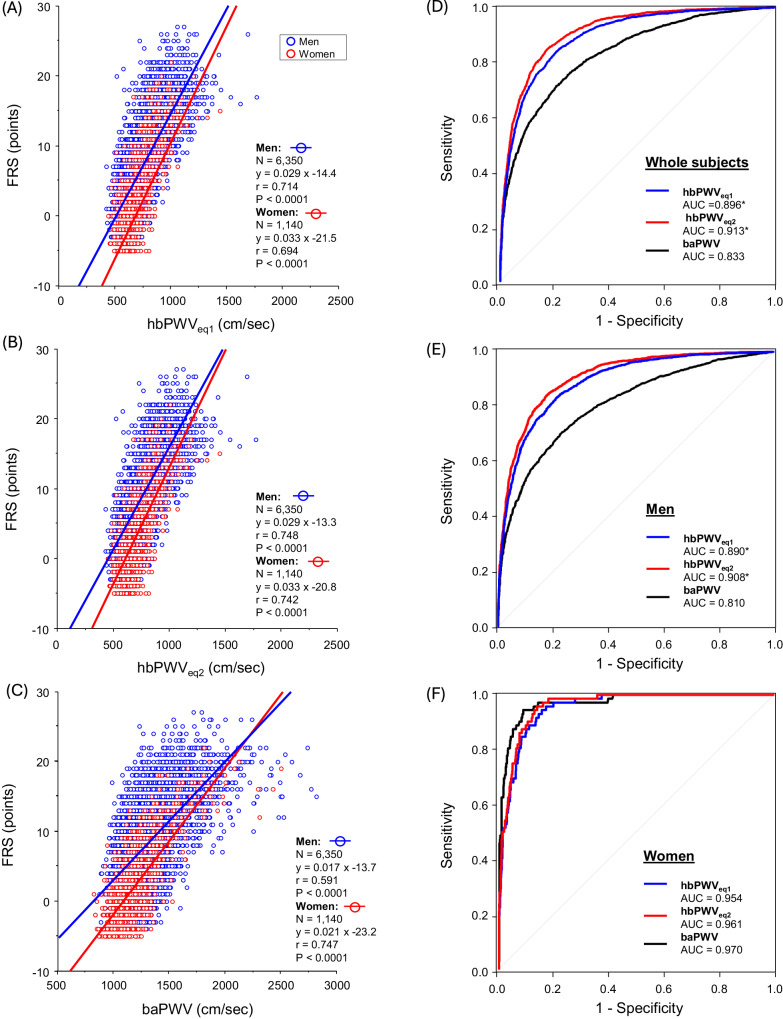
Simple correlation plots between pulse wave velocity (PWV) indices and Framingham risk score (FRS) in the cross-sectional study (Panel **A**: hbPWVe_q1_; Panel **B**: hbPWV_eq2_; Panel **C**: baPWV). Panels (**D**–**F**) indicate the results of a receiver operating characteristic (ROC) curve analysis on each PWV. * Significant difference in AUC of baPWV

Table 2 summarizes the results of a simple correlation analysis between PWVs and indices of CVD risks. In the cross-sectional study, PWVs significantly correlated with age, body mass index (BMI), heart rate, PEP/ET, BP, hyperlipidemi a- and diabetes mellitus-related blood markers. Hence, hbPWV_eq1_, hbPWV_eq2,_ and baPWV correlated with FRS in men (r = 0.714, 0.748, and 0.591, *P* < 0.0001 for all) and women (r = 0.694, 0.742, and 0.747, *P* < 0.0001 for all) (Fig. 3A–C). In the pooled participants, similar strong correlations were observed (r = 0.714, 0.749, and 0.643, *P* < 0.0001 for all). Because age was a strong covariate of both PWV and FRS even though it is included in the FRS calculation, we accounted for age in partial correlation analysis. These correlations remained significant even after accounting for age (hbPWV_eq1_: ρ = 0.260, hbPWV_eq2_: ρ = 0.269, baPWV: ρ = 0.402, *P* < 0.0001 for all). Furthermore, after adjusting for the use of drugs (i.e., anti-hypertension, hyperlipidemia, and diabetes mellitus), the correlations remained significant (hbPWV_eq1_: ρ = 0.666, hbPWV_eq2_: ρ = 0.707, baPWV: ρ = 0.578, ***P*** < 0.0001 for all).

**Table 2:** Correlation coefficients between pulse wave velocity and cardiovascular disease risk-related indices

	Cross-sectional study (*N* = 7490)			Longitudinal study (*N* = 3710)		
	hbPWV_eq1_	hbPWV_eq2_	baPWV	hbPWV_eq1_	hbPWV_eq2_	baPWV
Age	0.747	0.797	0.549	0.194	0.233	0.119
	*P* < 0.0001	*P* < 0.0001	*P* < 0.0001	*P* < 0.0001	*P* < 0.0001	*P* < 0.0001
BMI	0.099	0.105	0.188	–0.0164	–0.018	0.046
	*P* < 0.0001	*P* < 0.0001	*P* < 0.0001	*P* = 0.318	*P* = 0.276	*P* = 0.005
Heart rate	0.158	0.161	0.373	0.011	0.008	0.299
	*P* < 0.0001	*P* < 0.0001	*P* < 0.0001	*P* = 0.493	*P* = 0.626	*P* < 0.0001
PEP/ET	0.347	0.340	0.316	0.446	0.442	0.270
	*P* < 0.0001	*P* < 0.0001	*P* < 0.0001	*P* < 0.0001	*P* < 0.0001	*P* < 0.0001
Systolic BP	0.615	0.611	0.729	0.470	0.464	0.559
	*P* < 0.0001	*P* < 0.0001	*P* < 0.0001	*P* < 0.0001	*P* < 0.0001	*P* < 0.0001
Diastolic BP	0.698	0.693	0.715	0.473	0.466	0.487
	*P* < 0.0001	*P* < 0.0001	*P* < 0.0001	*P* < 0.0001	*P* < 0.0001	*P* < 0.0001
Total	0.209	0.213	0.229	0.070	0.072	0.092
cholesterol	*P* < 0.0001	*P* < 0.0001	*P* < 0.0001	*P* < 0.0001	*P* < 0.0001	*P* < 0.0001
LDL	0.142	0.149	0.144	0.030	0.031	0.027
cholesterol	*P* < 0.0001	*P* < 0.0001	*P* < 0.0001	*P* = 0.067	*P* = 0.059	*P* = 0.104
HDL	–0.054	–0.061	–0.095	0.053	0.060	0.065
cholesterol	*P* < 0.0001	*P* < 0.0001	*P* < 0.0001	*P* = 0.001	*P* = 0.0003	*P* < 0.0001
Triglyceride	0.184	0.186	0.251	0.037	0.034	0.071
	*P* < 0.0001	*P* < 0.0001	*P* < 0.0001	*P* = 0.025	*P* = 0.0376	*P* < 0.0001
Blood	0.299	0.312	0.345	–0.013	–0.012	0.072
glucose	*P* < 0.0001	*P* < 0.0001	*P* < 0.0001	*P* = 0.435	*P* = 0.453	*P* < 0.0001
HbA1C	0.254	0.264	0.297	–0.006	0.005	0.043
	*P* < 0.0001	*P* < 0.0001	*P* < 0.0001	*P* = 0.733	*P* = 0.763	*P* = 0.0083
FRS	0.714	0.749	0.643	0.216	0.225	0.229
	*P* < 0.0001	*P* < 0.0001	*P* < 0.0001	*P* < 0.0001	*P* < 0.0001	*P* < 0.0001

Correlation coefficients in the cross-sectional study indicate r values between PWVs and cardiovascular disease risk-related indices, whereas those in the longitudinal study indicate r values between changes in PWVs and cardiovascular disease risk-related indices. Abbreviations are shown in Table 1

As summarized in Supplementary Table 1, a stepwise regression analysis revealed that age and diastolic BP were major independent correlates of hbPWV_eq1_ and hbPWV_eq2_, explaining 69.3% and 74.6% of the total variance, whereas systolic BP and age were major independent correlates of baPWV, explaining 60% of the total variance.

A total of 2,399 participants were classified into the more than moderate general CVD risk group. As shown in Fig. 3D, baPWV stratified high CVD risk with 0.833 of the AUC. Compared with baPWV, hbPWV_eq1_ and hbPWV_eq2_ exhibited more robust ability to stratify general CVD risk in the whole subjects (AUC = 0.896 and 0.913, *P* < 0.0001 for both via DeLong’s test). Similar results were seen in men (Fig. 3E), whereas these AUC were comparable in women (Fig. 3F).

In the longitudinal study, changes in hbPWV correlated with corresponding changes in age, BP, PEP/ET, total cholesterol and triglyceride, and FRS (Table 2). A stepwise regression analysis revealed that changes in diastolic BP (β = 0.416, *P* < 0.0001) and PEP/ET (β = 0.353, *P* < 0.0001) were major independent correlates of changes in hbPWV, explaining 34.7% of the total variance, and were followed by changes in age, BMI, and total cholesterol as significant correlates, explaining additional 3.6% of the total variance (Supplementary Table 2). Regarding changes in baPWV, changes in systolic BP (β = 0.523, *P* < 0.0001) and PEP/ET (β = 0.172, *P* < 0.0001) were major independent correlates, explaining 35.6% of the total variance and were followed by changes in BMI, total cholesterol, and blood glucose as significant correlates, explaining additional 3.5% of the total variance.

**Fig. 4:**
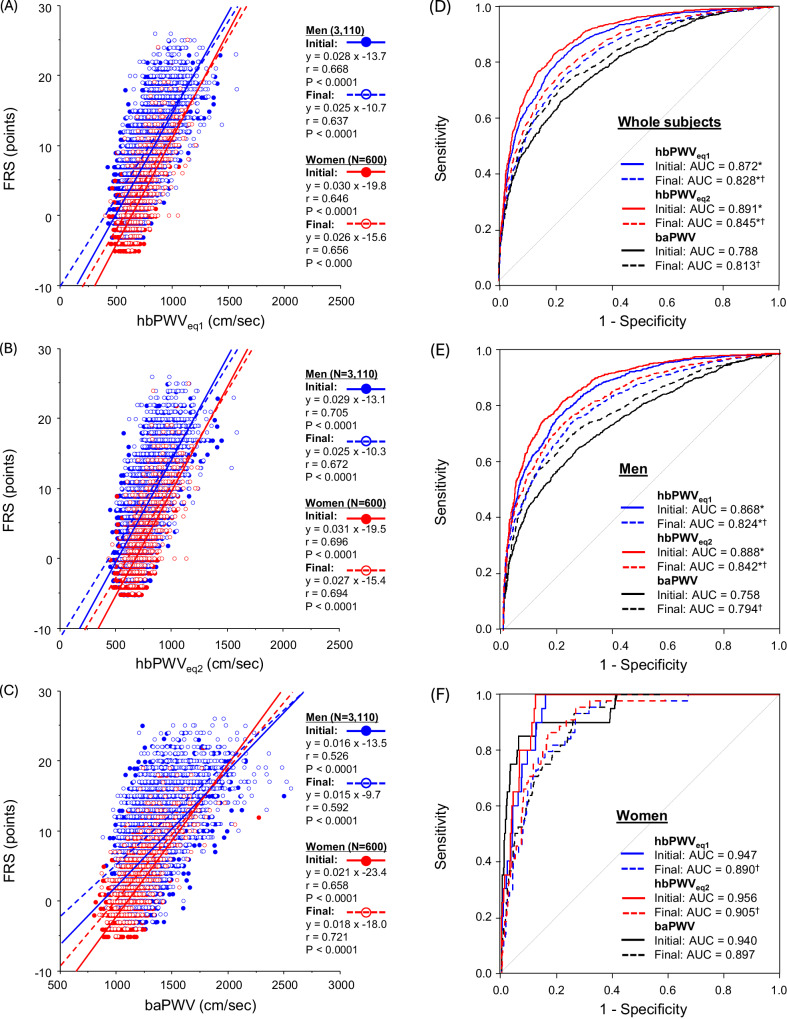
Simple correlation plots between pulse wave velocity (PWV) indices and Framingham risk score (FRS) in the longitudinal study (Panel **A**: hbPWVe_q1_; Panel **B**: hbPWV_eq2_; Panel **C**: baPWV). Panels (**D**–**F**) display the results of a receiver operating characteristic (ROC) curve analysis. *Significant difference vs. baPWV. ^†^Significant difference vs. the initial examination

Figure 4 displays the results of correlation and ROC curve analyses between PWVs and FRS at the initial and final examinations in the longitudinal observation. All PWVs correlated moderately with FRS at the initial and final examinations irrespective of sex (r = 0.526–0.721, *P* < 0.0001 for all, Figure A-C).

The more than moderate-risk holders were 1124 at the initial examination and 1740 at the last. Supplementary Table 3 summarizes the results of the ROC curve analysis. The AUC of hbPWVs significantly decreased from the initial to the final examination in the pooled subjects and men, whereas the AUC of baPWV increased (Figs. 4D, E). However, irrespective of the temporal dimension (e.g., the initial and final examinations), hbPWVs exhibited significantly higher AUC compared with baPWV. In women, the AUC of hbPWVs significantly decreased from the initial to the final examination. There were no significant differences in AUC among hbPWV_eq1_, hbPWV_eq2_, and baPWV at the initial and final examinations.

## Discussion

The salient findings of the present study are as follows. First, compared with baPWV, hbPWV exhibited stronger associations with age and FRS. Second, unlike baPWV that displayed gradually accelerated increases with advancing age, hbPWV demonstrated steady and consistent age-related increases throughout the lifespan. Third, the ROC curve analyses indicated that hbPWV depicted a more robust ability to stratify general cardiovascular disease (CVD) risk compared with baPWV. The results of the 10-year follow-up study were consistent with the findings of the cross-sectional investigation, irrespective of the temporal dimension (e.g., the initial and final examinations). The present cross-sectional and longitudinal study presents the data that further supports and substantiates the clinical relevance of hbPWV.

### Aging-related increases in PWVs

It is widely acknowledged that arterial stiffness increases with the progression of age [19–21, 37]. The majority of the currently available evidence stems from cross-sectional observations and does not capture intra-individual changes in arterial stiffness with aging. The present study is an attempt to close the gap in the field. When conventional correlation analysis is applied to non-independent observations such as repeated-measures aggregated data, biased results might emerge due to the violation of independence and/or differing patterns between subjects versus within subjects [35]. Therefore, we employed the repeated measures correlation (rmcorr), a statistical technique for determining the common within-individual association for paired measures assessed on more occasions for multiple individuals [35]. The rmcorr coefficients with age were substantially higher for hbPWV than for baPWV, irrespective of the arterial length equations with or without age. Additionally, we observed a relatively smaller variance of the aging slope for hbPWV across the entire lifespan. The longitudinal observations were consistent in finding linear and consistent age-related increases in hbPWV throughout the lifespan.

The age-related slope of baPWV exhibited attenuation in younger generations highlighting more pronounced increases after reaching middle age. This characteristic can be ascribed to the methodology employed in baPWV measurement. The arterial path length covered in baPWV encompasses both central elastic arteries (e.g., abdominal aorta) and peripheral muscular arteries (e.g., leg arteries). The stiffness of muscular arteries is generally greater than that of central elastic arteries in young and middle-aged adults and does not change much with aging [38, 39].

In contrast, central elastic arteries progressively stiffen from an early age ultimately surpassing the stiffness levels of muscular arteries around 50 years [6, 37]. This dynamic interplay engenders impedance matching between central arteries (such as the abdominal aorta) and peripheral conduit arteries (such as the iliac and femoral arteries) [40]. Such impedance matching, in turn, might mitigate wave reflection and promote the smooth forward transition of pulse waves in phase [3]. Consequently, this intricate mechanism is likely instrumental in the gradual escalation of baPWV with aging observed in older populations. Although the arterial trajectory of hbPWV also includes both elastic (i.e., the ascending aorta) and muscular (i.e., the subclavian, axial, and brachial arteries) arteries, the proportion of elastic arteries in this arterial trajectory is much greater. A previous study using MRI indicated a substantial decrease in distensibility of the ascending aorta even in younger age, whereas PWV of the aortic arch and the whole aorta do not show drastic changes with aging [5]. Additionally, in a cohort of the Framingham Heart Study, carotid-brachial PWV, which does not involve the ascending aorta, did not show an age-related increase [41]. In this study, hbPWV was measured only on the right arm due to the default settings of the automated cardiovascular screening device. However, this may have led to a more pronounced increase in hbPWV from early adulthood.

The correlation between age and hbPWV was much higher than that between age and cfPWV previous studies reported [20, 21]. Although we could not clarify this reason in this study, arterial path length estimation (e.g., no measurement), as well as a special skill-free, semi-automatic measurement, may reduce investigator-dependent measurement error and, hence, provide a high correlation coefficient.

### Relation to CVD risk

The elevated central arterial stiffness is linked to cardiovascular events and all-cause mortality [12–16]. Consequently, integrating assessments of arterial stiffness into routine clinical practice holds promise for enhancing and optimizing CVD risk management [42]. We [43] and others [44] substantiated that baPWV can act as a discriminator for identifying individuals at elevated risk of CVD. In the present study, we identified the linear and consistent age-related increases in hbPWV from the early life stage, implying the potential of a more sensitive marker for early detection of CVD risk. Additionally, hbPWV exhibited significant correlations with CVD risk markers such as BMI, brachial systolic and diastolic BP, LV systolic function (PEP/ET), hyperlipidemia- and diabetes mellitus-related blood markers. Accordingly, we determined the relationships between hbPWVs and FRS and whether hbPWVs could stratify moderate CVD risk (>10% of 10-year risk). hbPWV consistently demonstrated a superior AUC compared with baPWV, irrespective of the temporal dimension (e.g., the initial and final examinations). Furthermore, the AUC of hbPWV was higher at the initial than the final testing periods. These findings collectively indicate hbPWV as a more effective screening tool for ascertaining general CVD risk, especially in the early adult life stage.

While the precise mechanistic underpinnings of hbPWV’s superior sensitivity for early detection of CVD risk remain elusive, there are a number of supporting evidence. First, hbPWV mainly reflects proximal aortic stiffness, notably the most elastic segment directly linked to the LV [6]. Its role in attenuating cyclic mechanical forces generated by cardiac pulsations is particularly recognized. This critical segment is omitted in baPWV. Second, unlike baPWV, that is measured at the end of diastole via the foot-to-foot method, the pulse transition time for hbPWV is measured in systole and aligns with aortic pressure and LV afterload. Indeed, our previous study [6] revealed that the longitudinal changes in hbPWV exhibited a more robust correlation with corresponding alterations in aortic hemodynamic variables (e.g., aortic systolic blood pressure, augmentation pressure, and augmentation index), which are independent predictors of future CVD events and all-cause mortality [12, 45, 46].

Stepwise regression analyses applied to both cross-sectional and longitudinal studies showed that brachial diastolic BP was associated with hbPWV. This relation was observed in both central (e.g., aortic) and peripheral (e.g., brachial) sites [47]. The steady-state component of BP is characterized by mean arterial BP and diastolic BP is determined by cardiac output and peripheral resistance via Ohm’s law. In the community-based study involving older adults, total peripheral resistance appears to have the more dominant influence [48]. It is plausible that hbPWV may be influenced by factors that govern vascular resistance and hemodynamic load. Conversely, baPWV was found to be strongly influenced by brachial systolic BP [49].

### Study limitations

The study primarily comprised middle-aged men, focusing on individuals employed at a Japanese construction company. Despite the disproportionate representation of women, a substantial number of women were included in our cohort (1,200 in the cross-sectional study and 600 in the longitudinal study), ensuring sufficient statistical power. A sub-analysis examining the influence of sex on age-related changes in PWVs revealed no significant sex-related differences in the increase of hbPWV with age, suggesting that age-related increases in hbPWV were consistent in both men and women throughout early adulthood. Although the limited number of older participants prevented a comprehensive analysis of hbPWV changes in later life stages, at least hbPWV showed potential as a highly sensitive marker for CVD risk from early adulthood onwards.

There are additional limitations associated with the present study. First, this study was conducted as part of a company health check-up, and due to time constraints, cfPWV, the reference-standard assessment of large artery stiffness [50, 51], could not be measured. However, baPWV strongly and linearly correlates with cfPWV [6]. Unlike hbPWV, both cfPWV and baPWV do not include the stiffness of the proximal aorta. Therefore, they both may be characterizing age-related changes in the stiffness of the abdominal aorta. In this context, hbPWV may be seen as a complementary tool rather than a complete replacement for cfPWV. Second, following the health checkups, participants were advised to modify their lifestyle habits, including regular physical activity and dietary intake, during the observation. Given that we have no information or control over these individual choices, the potential impact of these variables on the observed alterations in PWVs remains unknown. Third, arterial path length for hbPWV and baPWV were not measured but estimated. However, cross-sectional and longitudinal observations consistently revealed that pulse transition times (i.e., Thb and Tba) are major determinants of PWVs, explaining ≈85% of the total variance. Thus, the contributions of arterial path length on PWVs were substantially smaller than that of the pulse transition time. Fourth, we did not explore the menopausal status (e.g., pre-, peri-, and post-menopause) or menstrual cycles of female participants. Furthermore, we lacked more detailed information regarding disease status (such as type I or II diabetes mellitus) and specific pharmacological treatments (including types of anti-hypertensive drugs). Last, the associations between hbPWV and clinical events or endpoints are unknown. The accuracy of predicting future events necessitates validation through prospective studies.

### Perspectives

hbPWV may reflect age-related changes in the proximal aortic properties, such as Windkessel function, starting from the early adult life stage. Consequently, this measure may offer distinct insights into arterial functions. baPWV has gained recognition as a convenient screening tool for hypertension-mediated organ damage [17, 18]. The Network for Research in Vascular Ageing recommended integrating assessment of sub-clinical arteriosclerosis (via baPWV), advanced atherosclerosis (via ABI and systolic interarm blood pressure difference), and central hemodynamics associated with LV afterload (via pulse wave analysis) to gain a holistic snapshot of a cardiovascular health management [42]. Therefore, incorporating hbPWV measurement into this framework enables a more comprehensive clinical assessment and management of CVD.

### Perspective of Asia

Many Asian countries are experiencing rapid growth of the aging population [52]. As stated in the aphorism “you are as old as your artery”, arterial stiffness is an indicator of biological aging and can have substantial public health implications for Asian populations [53]. Unknown to most, the world’s first automated commercial device for measuring pulse wave velocity was manufactured and sold in Japan in 1968 [54]. A measure of arterial stiffness has not been incorporated in routine clinical practice worldwide. However, Asian countries including Japan are the first to fully integrate the measure of arterial stiffness in routine clinical medicine. This success is mainly attributed to the development of baPWV [49] and the cardio-ankle vascular index (CAVI) [55]. hbPWV evaluated in the present study is one of various measures of arterial stiffness that are obtained simultaneously when baPWV and CAVI are measured. However, this particular measure had been largely ignored. As indicated by our present findings, hbPWV may be a promising measure of arterial stiffness that may improve the early detection capabilities of CVD risk.

## Conclusion

To further evaluate the promising modality of hbPWV in evaluating central elastic artery stiffness, we conducted combined cross-sectional and longitudinal analyses to determine age-associated changes and general CVD risk. Compared with baPWV, hbPWV demonstrated more robust associations with age and FRS. Particularly noteworthy was the feature of hbPWV to display significantly greater age-related increases even in young adults. In contrast, baPWV exhibited a gradually accelerated increment with advancing age. The ROC curve analysis further substantiated the superior efficacy of hbPWV in stratifying general CVD risk compared with baPWV. Taken together, our present findings suggest that hbPWV undergoes a linear augmentation with age, commencing from an early life stage onward, rendering it a potential marker for discerning CVD risk.

## Supplementary information

Supplementary Tables

Supplementary Figure 1
