# Supplementary material for: Cross-sectional and longitudinal evaluation of heart-to-brachium pulse wave velocity for cardiovascular disease risk
Source: Hypertens Res. 2024 Aug 1;47(11):3010–24. doi: 10.1038/s41440-024-01805-5 (PMC11534680; doi:10.1038/s41440-024-01805-5)
Supplement: Supplementary file 1 — Supplementary Tables [file 41440_2024_1805_MOESM1_ESM.docx]

**Supplemental Table 1. Results of multivariate regression analysis to determine independent correlates of heart-brachium and brachial-ankle pulse wave velocity in the cross-sectional study.**

|  | ***Dependent variable: hbPWV_eq1_*** | | | | | | |
| --- | --- | --- | --- | --- | --- | --- | --- |
|  |  | β | Normalized β | Multiple R | Multiple R^2^ | R^2^ change | P-value |
| 1 | Age | 7.143 | 0.503 | 0.747 | 0.558 | 0.558 | P<0.0001 |
| 2 | DBP | 6.072 | 0.491 | 0.833 | 0.693 | 0.135 | P<0.0001 |
| 3 | PEP/ET | 466.656 | 0.158 | 0.845 | 0.713 | 0.020 | P<0.0001 |
| 4 | HR | -2.121 | -0.146 | 0.854 | 0.729 | 0.016 | P<0.0001 |
| 5 | BMI | -6.062 | -0.137 | 0.862 | 0.743 | 0.014 | P<0.0001 |
| 6 | HbA1C | 10.210 | 0.045 | 0.863 | 0.744 | 0.001 | P<0.0001 |
| 7 | TG | 0.044 | 0.031 | 0.863 | 0.745 | 0.001 | P<0.0001 |
| 8 | TC | -0.090 | -0.021 | 0.863 | 0.745 | 0.000 | P<0.0001 |
|  | |  |  |  |  |  |  |
|  | ***Dependent variable: hbPWV_eq2_*** | | | | | | |
|  |  | β | Normalized β | Multiple R | Multiple R^2^ | R^2^ change | P-value |
| 1 | Age | 8.783 | 0.583 | 0.797 | 0.635 | 0.635 | P<0.0001 |
| 2 | DBP | 4.295 | 0.328 | 0.864 | 0.746 | 0.111 | P<0.0001 |
| 3 | PEP/ET | 496.499 | 0.159 | 0.874 | 0.764 | 0.018 | P<0.0001 |
| 4 | HR | -2.100 | -0.135 | 0.882 | 0.778 | 0.014 | P<0.0001 |
| 5 | BMI | -6.071 | -0.129 | 0.8883 | 0.789 | 0.011 | P<0.0001 |
| 6 | HbA1C | 9.178 | 0.038 | 0.8887 | 0.790 | 0.001 | P<0.0001 |
| 7 | TG | 0.046 | 0.030 | 0.8890 | 0.790 | 0.000 | P<0.0001 |
| 8 | TC | -0.125 | -0.028 | 0.8893 | 0.791 | 0.001 | P<0.0001 |
|  | |  |  |  |  |  |  |
|  | ***Dependent variable: baPWV*** | | | | | | |
|  | | β | Normalized β | Multiple R | Multiple R^2^ | R^2^ change | P-value |
| 1 | SBP | 7.473 | 0.563 | 0.729 | 0.531 | 0.531 | P<0.0001 |
| 2 | Age | 5.430 | 0.254 | 0.776 | 0.603 | 0.072 | P<0.0001 |
| 3 | PEP/ET | 524.353 | 0.118 | 0.788 | 0.621 | 0.019 | P<0.0001 |
| 4 | HR | 2.075 | 0.095 | 0.794 | 0.631 | 0.010 | P<0.0001 |
| 5 | BMI | -6.847 | -0.103 | 0.798 | 0.636 | 0.005 | P<0.0001 |
| 6 | Glu | 0.571 | 0.044 | 0.801 | 0.642 | 0.005 | P<0.0001 |
| 7 | TG | 0.129 | 0.059 | 0.803 | 0.645 | 0.004 | P<0.0001 |
| 8 | HbA1C | 13.907 | 0.041 | 0.804 | 0.646 | 0.001 | P<0.0001 |

baPWV, brachial-ankle pulse wave velocity; BMI, body mass index; DBP, diastolic blood pressure; Glu, blood glucose; HbA1C, hemoglobin A1C; hbPWV, heart-brachium pulse wave velocity; HR, heart rate; PEP/ET, the ratio of the pre-ejection period to ejection time; TC, total cholesterol; TG, triglyceride; SBP, systolic blood pressure.

**Supplemental Table 2. Results of multivariate regression analysis to determine independent correlates of changes in heart-brachium and brachial-ankle pulse wave velocity in the longitudinal study.**

|  | ***Dependent variable: ΔhbPWV_eq1_*** | | | | | | |
| --- | --- | --- | --- | --- | --- | --- | --- |
|  | | β | Normalized β | Multiple R | Multiple R^2^ | R^2^ change | P-value |
| 1 | ΔDBP | 4.576 | 0.416 | 0.473 | 0.224 | 0.224 | P<0.0001 |
| 2 | ΔPEP/ET | 764.611 | 0.353 | 0.589 | 0.347 | 0.123 | P<0.0001 |
| 3 | ΔAge | 8.190 | 0.165 | 0.610 | 0.372 | 0.025 | P<0.0001 |
| 4 | ΔBMI | -4.879 | -0.077 | 0.616 | 0.380 | 0.008 | P<0.0001 |
| 5 | ΔTC | -0.206 | -0.062 | 0.619 | 0.383 | 0.003 | P<0.0001 |

|  | ***Dependent variable: ΔhbPWV_eq2_*** | | | | | | |
| --- | --- | --- | --- | --- | --- | --- | --- |
|  | | β | Normalized β | Multiple R | Multiple R^2^ | R^2^ change | P-value |
| 1 | ΔDBP | 1.045 | 0.096 | 0.466 | 0.217 | 0.217 | P<0.0001 |
| 2 | ΔPEP/ET | 813.761 | 0.380 | 0.582 | 0.338 | 0.121 | P<0.0001 |
| 3 | ΔAge | 10.880 | 0.222 | 0.615 | 0.378 | 0.040 | P<0.0001 |
| 4 | ΔSBP | 2.866 | 0.363 | 0.649 | 0.421 | 0.043 | P<0.0001 |
| 5 | ΔTC | -0.293 | -0.090 | 0.654 | 0.428 | 0.007 | P<0.0001 |

|  | ***Dependent variable: ΔbaPWV*** | | | | | | |
| --- | --- | --- | --- | --- | --- | --- | --- |
|  |  | β | Normalized β | Multiple R | Multiple R^2^ | R^2^ change | P-value |
| 1 | ΔSBP | 6.012 | 0.523 | 0.559 | 0.312 | 0.312 | P<0.0001 |
| 2 | ΔPEP/ET | 537.539 | 0.172 | 0.596 | 0.356 | 0.044 | P<0.0001 |
| 3 | ΔBMI | 8.910 | 0.125 | 0.608 | 0.370 | 0.014 | P<0.0001 |
| 4 | ΔTC | 2.276 | 0.134 | 0.620 | 0.385 | 0.015 | P<0.0001 |
| 5 | ΔGlu | -5.313 | -0.058 | 0.624 | 0.390 | 0.005 | P<0.0001 |

Abbreviations are presented in Supplemental Table 1.

**Supplemental Table 3. Results of Receiver Operating Characteristic (ROC) curve analysis.**

|  |  | AUC | 95% CI | | | Cut-off | Sensitivity | Specificity |
| --- | --- | --- | --- | --- | --- | --- | --- | --- |
| **Cross-sectional study** | | | | | | | | |
| Whole subjects | hbPWV_eq1_ | 0.896* | 0.889 | - | 0.904 | 777 | 0.839 | 0.796 |
|  | hbPWV_eq2_ | 0.913* | 0.907 | - | 0.920 | 755 | 0.853 | 0.824 |
|  | baPWV | 0.833 | 0.823 | - | 0.843 | 1,324 | 0.714 | 0.795 |
| Men | hbPWV_eq1_ | 0.890* | 0.882 | - | 0.899 | 777 | 0.835 | 0.786 |
|  | hbPWV_eq2_ | 0.908* | 0.901 | - | 0.916 | 757 | 0.845 | 0.820 |
|  | baPWV | 0.810 | 0.798 | - | 0.821 | 1,324 | 0.706 | 0.768 |
| Women | hbPWV_eq1_ | 0.954 | 0.937 | - | 0.971 | 781 | 0.944 | 0.845 |
|  | hbPWV_eq2_ | 0.961 | 0.947 | - | 0.976 | 757 | 0.958 | 0.861 |
|  | baPWV | 0.970 | 0.953 | - | 0.987 | 1,340 | 0.944 | 0.912 |
|  |  |  |  |  |  |  |  |  |
| ***Longitudinal-Initial exam*** | | | | | | | | |
| Whole subjects | hbPWV_eq1_ | 0.872* | 0.860 | - | 0.884 | 761 | 0.799 | 0.787 |
|  | hbPWV_eq2_ | 0.891* | 0.880 | - | 0.902 | 727 | 0.831 | 0.795 |
|  | baPWV | 0.788 | 0.772 | - | 0.804 | 1,299 | 0.656 | 0.774 |
| Men | hbPWV_eq1_ | 0.868* | 0.855 | - | 0.881 | 769 | 0.770 | 0.802 |
|  | hbPWV_eq2_ | 0.888* | 0.876 | - | 0.899 | 753 | 0.762 | 0.852 |
|  | baPWV | 0.758 | 0.740 | - | 0.776 | 1,299 | 0.652 | 0.737 |
| Women | hbPWV_eq1_ | 0.947 | 0.921 | - | 0.972 | 771 | 1.000 | 0.841 |
|  | hbPWV_eq2_ | 0.956 | 0.934 | - | 0.977 | 757 | 1.000 | 0.878 |
|  | baPWV | 0.940 | 0.886 | - | 0.993 | 1,349 | 0.850 | 0.938 |
|  |  |  |  |  |  |  |  |  |
| ***Longitudinal-Final exam:*** | | | | | | | | |
| Whole subjects | hbPWV_eq1_ | 0.828* | 0.815 | - | 0.841 | 817 | 0.748 | 0.761 |
|  | hbPWV_eq2_ | 0.845* | 0.833 | - | 0.857 | 799 | 0.764 | 0.770 |
|  | baPWV | 0.813 | 0.799 | - | 0.826 | 1,322 | 0.726 | 0.754 |
| Men | hbPWV_eq1_ | 0.824* | 0.809 | - | 0.838 | 816 | 0.748 | 0.757 |
|  | hbPWV_eq2_ | 0.842* | 0.829 | - | 0.856 | 806 | 0.740 | 0.791 |
|  | baPWV | 0.794 | 0.778 | - | 0.809 | 1,332 | 0.705 | 0.755 |
| Women | hbPWV_eq1_ | 0.890 | 0.849 | - | 0.931 | 800 | 0.932 | 0.734 |
|  | hbPWV_eq2_ | 0.905 | 0.869 | - | 0.941 | 825 | 0.864 | 0.833 |
|  | baPWV | 0.897 | 0.861 | - | 0.933 | 1,264 | 0.932 | 0.737 |

AUC, area under the ROC curve; CI, confidential interval. * Significant difference in AUC of ROC curve vs. that of baPWV of the same category.
