# Supplementary figures and images for: Cross-sectional and longitudinal evaluation of heart-to-brachium pulse wave velocity for cardiovascular disease risk
Source: Hypertens Res. 2024 Aug 1;47(11):3010–24. doi: 10.1038/s41440-024-01805-5 (PMC11534680; doi:10.1038/s41440-024-01805-5)

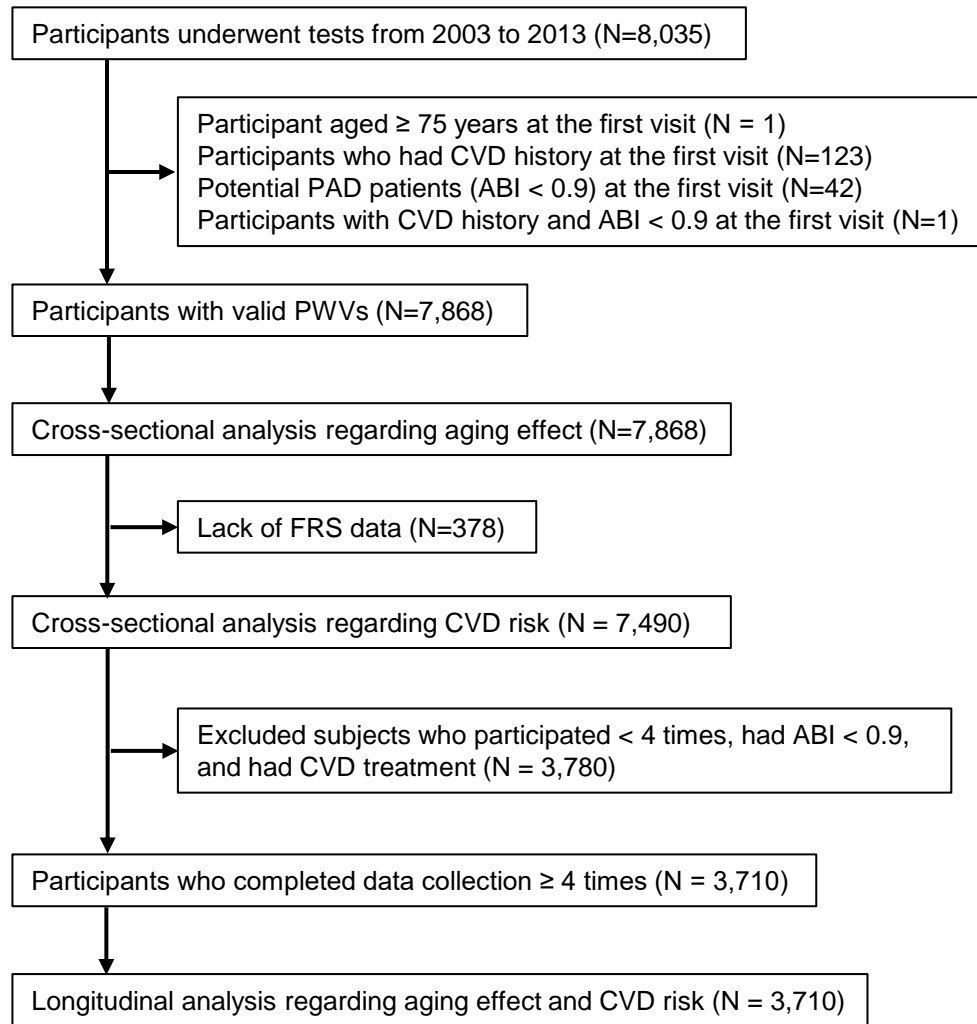

Supplemental figure 1. The schematic representation of the participant selection process.

Supplement: Supplementary file 2 — Supplementary Figure 1 [file 41440_2024_1805_MOESM2_ESM.pdf]
